# Identifying configurations of behavior change techniques in effective medication adherence interventions: a qualitative comparative analysis

**DOI:** 10.1186/s13643-016-0255-z

**Published:** 2016-05-04

**Authors:** Leila Kahwati, Meera Viswanathan, Carol E. Golin, Heather Kane, Megan Lewis, Sara Jacobs

**Affiliations:** RTI International, Research Triangle Park, NC USA; Departments of Medicine and Health Behavior, University of North Carolina, Chapel Hill, NC USA

**Keywords:** Medication adherence, Qualitative comparative analysis, Behavior change, Systematic review

## Abstract

**Background:**

Interventions to improve medication adherence are diverse and complex. Consequently, synthesizing this evidence is challenging. We aimed to extend the results from an existing systematic review of interventions to improve medication adherence by using qualitative comparative analysis (QCA) to identify necessary or sufficient configurations of behavior change techniques among effective interventions.

**Methods:**

We used data from 60 studies in a completed systematic review to examine the combinations of nine behavior change techniques (increasing knowledge, increasing awareness, changing attitude, increasing self-efficacy, increasing intention formation, increasing action control, facilitation, increasing maintenance support, and motivational interviewing) among studies demonstrating improvements in adherence.

**Results:**

Among the 60 studies, 34 demonstrated improved medication adherence. Among effective studies, increasing patient knowledge was a necessary but not sufficient technique. We identified seven configurations of behavior change techniques sufficient for improving adherence, which together accounted for 26 (76 %) of the effective studies. The intervention configuration that included increasing knowledge and self-efficacy was the most empirically relevant, accounting for 17 studies (50 %) and uniquely accounting for 15 (44 %).

**Conclusions:**

This analysis extends the completed review findings by identifying multiple combinations of behavior change techniques that improve adherence. Our findings offer direction for policy makers, practitioners, and future comparative effectiveness research on improving adherence.

**Electronic supplementary material:**

The online version of this article (doi:10.1186/s13643-016-0255-z) contains supplementary material, which is available to authorized users.

## Background

Medication adherence is a complex behavior with multiple determinants that vary among individuals. Although there is only one way for patients to be perfectly adherent, there are many ways that patients can be nonadherent, such as missing doses, taking doses late, taking fewer pills at each dose, or stopping a regimen early. Poor medication adherence is associated with increased morbidity, mortality, and costs across a range of clinical conditions [1]. The World Health Organization estimated that adherence to long-term therapy for chronic conditions is 50 % in developed countries and even lower in developing countries [2]. The importance of medication adherence and the variability in nonadherence illustrates the need for methods that can pinpoint specific and effective intervention components to enhance adherence.

Similar to other health behaviors, interventions to improve medication adherence are diverse and complex and often use combinations of behavior change techniques (BCTs), for example, techniques to increase self-efficacy or change attitudes. Some members of our study team [M.V., C.G] recently completed an Agency for Healthcare Quality and Research (AHRQ)-sponsored review of interventions to improve medication adherence among outpatients prescribed long-term medication therapy for chronic conditions [3, 4]. This review, which included 67 studies overall, synthesized findings first by clinical condition and then by intervention type, such as case management, self-management support, collaborative care, or patient education, resulting in 40 different strength of evidence grades for each small group of studies that used the same intervention type among a study population defined by clinical condition. Heterogeneity among the included studies precluded quantitative synthesis (i.e., meta-analysis). The most consistent evidence of improvement in medication adherence across clinical conditions was for interventions that included case management and educational interventions. Within clinical conditions, the strongest support was found for self-management of medications for short-term improvement in adherence for asthma patients; collaborative care or case management programs for short-term improvement of adherence and to improve symptoms for patients taking depression medications; and pharmacist-led approaches for hypertensive patients to improve systolic blood pressure. However, this synthesis did not evaluate the effectiveness of the various BCTs used across this body of evidence.

Interest is emerging in the use of qualitative comparative analysis (QCA) within systematic reviews of complex interventions because of the significant clinical heterogeneity faced when trying to synthesize such evidence [5]. QCA originates from the comparative social sciences to study complex phenomena. QCA uses set theory—a branch of mathematical logic that studies the properties of sets, which are well-defined collections of objects—to examine relationships between configurations of conditions (c.f., explanatory variables) and an outcome. QCA is a non-probabilistic method that may be useful for identifying complex causal patterns that variable-oriented methods may miss and it is an approach that may capitalize on the heterogeneity within reviews that typically limits quantitative synthesis [6–9]. Relationships of necessity (an explanatory variable or combination of variables is always present when the outcome is present) and sufficiency (the outcome is always present when the explanatory variable or combination of variables is present) are two examples of complex causal patterns that QCA can be used to identify. For medication adherence interventions, QCA offers a novel way to make sense of the underlying variation in population, intervention components, and context and relationship to intervention effectiveness.

In the present paper, we present findings from our use of QCA to identify the combinations (i.e., configurations) of patient-directed BCTs that were necessary or sufficient, or both, for improving medication adherence across the diverse body of evidence in the AHRQ review previously completed by members of our study team. This analysis was part of a larger study to examine the suitability of using QCA within a systematic review context. We expected that using QCA could elucidate combinations of patient-directed BCTs that, in turn, could inform both current practice and intervention design for future comparative effectiveness research.

## Methods

We describe our approach to using QCA within the completed AHRQ systematic review in a companion article and figure also published in this issue [10]. The companion article provides a more in-depth discussion of the method, a simple hypothetical example and glossary of terms commonly used in reporting this method, and discussion of how this method can be applied within a systematic review context. We applied existing standards of practice for conducting QCA, though methods are evolving as with any relatively novel method [11].

### Case identification

We used the studies in the completed AHRQ systematic review of interventions to improve medication adherence as cases. The completed review was conducted by members of our study team using methods associated with the AHRQ Effective Health Care Program (available at http://www.ncbi.nlm.nih.gov/books/NBK47095/), and results from this review were published as an evidence report and summarized in a peer-reviewed journal-length article [3, 4]. This review was limited to studies in the USA in adults with chronic conditions, excluding HIV/AIDS, severe mental illness, and substance abuse. Studies were included if they intended to improve adherence with self-administered, prescribed medication; example interventions included patient education, counseling, behavioral interventions, case management, reminders, and shared decision-making. The completed review included a total of 67 studies, but we excluded 5 studies from the QCA that evaluated policy-level interventions (e.g., copayment elimination) because these studies lacked patient-directed BCTs. We also excluded 2 other studies; one that did not include any patient-directed element and one that evaluated an intervention involving medication packaging because it was too dissimilar from BCTs included in all of the other studies [12, 13]. Consequently, we included 60 studies, all randomized controlled trials assessed as having low or medium risk of bias, as cases in the QCA.

### Outcome set calibration and set membership value assignment

We specified that a study would be included in the outcome set if the intervention group demonstrated an improvement in medication adherence. Studies in the review used a variety of adherence outcomes measured at various time points based on self-report, prescription fills and refills, or medication event monitoring systems (e.g., medication bottles with a microchip that registers the date and time of every bottle opening). In the absence of a common outcome used across all studies, we considered studies for which the intervention group demonstrated statistically significant improvements on at least one measure of adherence as compared with a usual care group to be fully “in” the outcome set of effective studies (set membership value of “1.”). Studies without improvement on at least one adherence outcome were assigned as fully “out” of the set of effective studies (set membership value of “0.”).

### Condition set calibration and set membership value assignment

We specified nine BCT condition sets and defined that a study would be included in each BCT set based on whether or not the specified BCT was a feature of the study intervention. The BCTs specified for abstraction in the completed review were derived from an existing meta-analysis of medication adherence interventions and a published taxonomy of BCTs [14, 15]. The nine BCTs we included in the QCA were techniques to increase *knowledge*, *awareness*, *self-efficacy*, *intention formation*, techniques to influence *action control*, *attitude*, *maintenance*; or were techniques that used *facilitation*, or *motivational interviewing* strategies. A brief description of each BCT is in Table 1. Although the completed review abstracted data for 12 BCTs, we did not include 3 BCTs—social influence, contingent reward, and stress management—in the QCA because they were infrequently used.

**Table 1:** Behavior change techniques (BCTs) used in interventions to improve medication adherence [14, 15]

Behavior change technique (abbreviation for analysis^a^)	Description
Knowledge (K)	General information about behavior-related health consequences, use of individualized information, increase in understanding/memory enhancement
Awareness (R)	Risk communication, self-monitoring, reflective listening, behavioral feedback
Attitudes (T)	Targets attitudes toward behavior
Self-efficacy (S)	Modeling, practice/skills training, verbal persuasion, coping response, graded tasks, reattribution of success/failure
Intention formation (I)	General intention, medication schedule, goals, behavioral contract
Action control (C)	Cues/reminders, self-persuasion, social support
Maintenance (M)	Maintenance goals, relapse prevention
Facilitation (F)	Ongoing professional support, dealing with adverse effects, individualizing/simplifying regimen (fewer pills, fewer medications, less frequent dosing, timing of dosing to fit individual schedule), reducing environmental barriers
Motivational interviewing (G)	Client-centered yet directive counseling style that facilitates behavior change through helping clients resolve ambivalence.

^a^Abbreviations in parentheses are used in the presentation of results in Table 3

During the review process, each study was evaluated to identify whether or not it used each of the BCTs of interest based on information provided in the article and standard definitions of each BCT compiled by the review team. Abstracted data from these studies could indicate that a study used more than one BCT. We used this information abstracted during the review to determine whether or not a BCT was a feature of the study intervention for the QCA. For each of the BCT sets, we assigned a set membership value of “1” if the study used the BCT and assigned a set membership value of “0” if the study did not use the BCT or if it was coded as “unclear” by the study abstractors.

### Analysis

Because we calibrated sets dichotomously, we used crisp-set QCA. Using Stata Release 13 (StataCorp, College Station, TX), we generated 2 × 2 contingency tables using each BCT and outcome set membership values to identify individually necessary and sufficient BCTs for the outcome of improved medication adherence. A BCT is considered necessary if it is consistently present as a feature of studies demonstrating improved adherence, whereas a BCT is considered sufficient if improved medication adherence is consistently present when the BCT is present. We used a consistency threshold of 90 % for necessity and 80 % for sufficiency in our analyses.

Next, we used BCT set and outcome set membership values assigned to create a truth table, which is the analytic device used in QCA to evaluate the necessity and sufficiency of configurations of BCTs. A truth table includes all 2^k^ logically possible configurations of condition set membership values, where *k* is equal to the number of condition sets included in the analysis. Our analysis with nine BCT sets generates a truth table with 512 rows, and we assigned each study in our analysis to the truth table row that represented its configuration of BCT set membership values. We then determined the consistency for the truth table row based on the outcome set membership values for the studies in the row. If the proportion of studies within a truth table row demonstrating improved adherence is above the specified consistency threshold (80 % in our analysis), the outcome value for that row is assigned a value of “1”; all other rows are assigned a value of “0.”

Each row with an outcome set value of “1” represents a sufficient configuration of BCTs for the outcome of improved adherence. We used fsQCA version 2.5 to perform logical minimization of sufficient truth table rows and to calculate parameters of fit (consistency, raw coverage, unique coverage) [16]. Lastly, we informed our interpretation of the QCA solutions generated by examining how they were represented both within single studies and across studies.

## Results

### Individually necessary and sufficient BCTs

Among the 60 studies, 34 demonstrated improved medication adherence. Among these studies, no individual BCT was both necessary (a BCT that is always present when the outcome is present) and sufficient (outcome always present when the BCT is present) for improved medication adherence. Increasing *knowledge* was the only necessary individual BCT for improved adherence; it was present in 31 of the 34 studies (consistency 91 %). No other individual BCT was near the threshold for necessity (Table 2). Two BCTs were identified as individually sufficient: enhancing *self-efficacy* (consistency 90 %) and improving *attitude* (consistency 83 %). Another BCT, *motivational interviewing* (consistency 78 %), was close to the consistency threshold. A detailed discussion of consistency is provided in the companion article in this issue [10].

**Table 2:** Necessity and sufficiency of individual behavior change techniques (BCTs) used within studies demonstrating improved medication adherence

Behavior change technique (BCT) (abbreviation for analysis^a^)			Necessity	Sufficiency
	Number of studies with BCT	Number of studies with BCT from among those studies demonstrating improved adherence (*N* = 34)^b^	Of studies demonstrating improved adherence (*N* = 34)^b^, percent with BCT	Of studies with BCT, percent that demonstrate improved adherence
Knowledge (K)	53	31	91 %	58 %
Facilitation (F)	32	16	47 %	50 %
Awareness (R)	29	15	44 %	52 %
Self-efficacy (S)	20	18	53 %	90 %
Intention formation (I)	14	9	26 %	64 %
Action control (C)	10	5	15 %	50 %
Attitude (T)	12	10	29 %	83 %
Maintenance (M)	9	5	15 %	56 %
Motivational interviewing (G)	9	7	21 %	78 %

^a^Abbreviations used in parentheses are used in the presentation of results in Table 3

^b^34 studies demonstrated improved medication adherence out of a total of 60 used for this analysis

### Sufficient configurations of BCTs and solutions

We identified 37 unique configurations of BCTs (i.e., truth table rows) present among the 60 studies; this represents 7 % of the 512 truth table rows comprising all logically possible configurations. Of these 37 rows, 19 had a consistency higher than our specified threshold (80 %) for the outcome of improved medication adherence; these rows were used for subsequent truth table analyses. The rows not used included 14 that had an outcome value of “0” for improved medication adherence, and 4 that were contradictory, with outcome consistency ranging from 33 to 50 %. The rows without any empirical cases are referred to as logical remainders and were used in generating results as described in the next paragraph. The truth table is provided in the online supplementary material (Additional file 1: Appendix A).

Findings from the logical minimization of the truth table are presented as solutions. The complex solution—the solution that makes no simplifying assumptions about logical remainders—identified 14 sufficient configurations of BCTs within studies demonstrating improved medication adherence. The parsimonious solution—the solution generated automatically by the software using simplifying assumptions about logical remainders to achieve the most parsimonious results without evaluating the plausibility of those assumptions—identified 5 sufficient configurations. The intermediate solution—the solution generated using researcher knowledge and expectations to guide the simplifying assumptions made by the software—identified 7 sufficient configurations. We present the intermediate solution as our main finding because we believe that some of the simplifying assumptions made to achieve maximum parsimony are substantively implausible. Additional detail about the parsimonious and complex solutions and additional analyses related to identifying configurations of BCTs in studies without improvements in medication adherence are provided in the online supplementary material (Additional file 1: Appendix B and C, respectively).

The intermediate solution and its parameters of fit are detailed in Table 3. In this table, sufficient configurations of BCTs are represented by single letter abbreviations where uppercase notation represents the presence of the BCT and lowercase notation represents the absence of the BCT. The solution consistency was 100 %, and solution coverage was 76 %, with 26 of the 34 effective studies accounted for. Three studies were covered by more than one BCT configuration and five of the configurations uniquely covered at least one study. The BCT configuration consisting of increasing *knowledge* AND enhancing *self-efficacy* (“KS”) covered half of the studies demonstrating improved medication adherence (17 studies, raw coverage 50 %) and uniquely covered all but two of those studies (unique coverage 44 %). The other six configurations had raw coverage ranging from one study (3 %) to four studies (12 %). We tested different consistency thresholds between 70 and 90 % and did not find any differences in the results (not shown).

**Table 3:** Intermediate solution for configurations of behavior change techniques (BCTs) used in effective interventions to improve medication adherence

Solution parameters				
Consistency^a^: 100 % (34 studies) Coverage^b^: 76 % total (26 studies) 68 % unique (23 studies) 8 % overlapping (3 studies) Truth table rows covered: 7.2 % (37 of 512 rows)			Number of studies with outcome not covered^c^: 8 Bender et al. 2010 [46], Hoffman et al. 2003 [47], Lee et al. 2006 [48], Okeke et al. 2009 [49], Ross et al. 2004 [50], Smith et al. 2008 [51], Solomon et al. 1998 [52], Waalen et al. 2009 [53]	
Configuration^d^	Consistency (%)	Raw coverage % (# of cases)	Unique coverage % (# of cases)	Study
KS	100	50 (17)	44 (15)	^e^Berg et al. 1997 [24], ^e^Janson et al. 2003 [18], ^e^Janson et al. 2009 [17], ^e^Johnson et al. 2006a [29], ^e^Johnson et al, 2006b [28], ^e^Katon et al. 1995 [26], ^e^Katon et al. 1999/Katon et al. 2002 [25], ^e^Katon et al. 1996 [21], Katon et al. 2001/Ludman et al. 2003/Van Korff et al. 2003 [22], ^e^Murray et al. 2007 [23], Ogedegbe et al. 2012 [54], ^e^Rudd et al. 2004 [19], ^e^Schaffer et al. 2004 [33], ^e^Simon et al. 2004 [30], ^e^Stacy et al. 2009 [32], ^e^Wilson et al. 2010 [20], ^e^Wu et al. 2012 [31]
fG	100	12 (4)	6 (2)	^e^Berger et al. 2005 [35], ^e^Friedman et al. 1996 [34], Katon et al. 2001 [22], Ogedegbe et al. 2012 [54]
rSIT	100	6 (2)	0 (0)	Ogedegbe et al. 2012 [54], Wolever et al. 2010 [55]
kfCm	100	3 (1)	3 (1)	^e^Fulmer et al. 1999 [43]
fSmIT	100	6 (2)	0 (0)	Ogedegbe et al. 2012 [54], Wolever et al. 2010 [55]
KRFICm	100	6 (2)	6 (2)	^e^Bosworth et al. 2008 [37], ^e^Rich et al. 1996 [38]
KrFT	100	9 (3)	9 (3)	^e^Bogner et al. 2012 [40], ^e^Bogner et al. 2010 [39], ^e^Bogner et al. 2008 [41]

^a^Consistency is determined by dividing the number of studies in the outcome set that are covered by the configuration by the number of studies covered by the configuration. Consistency can range from 0 to 100 %. A consistency of 100 % indicates that all studies covered by the configuration are also in the outcome set (i.e., had improved adherence)

^b^Total coverage is determined by dividing the number of studies covered by any sufficient configuration in the solution by the number of studies demonstrating improved adherence. Unique coverage is determined by dividing the number of studies that are only covered by one of the sufficient configurations by the total number of studies demonstrating improved adherence. Total and unique coverage can range from 0 to 100 %. Overlapping coverage is the difference between total and unique coverage

^c^Studies with the outcome that are not covered by a configuration (i.e., unexplained cases) are studies that were located in contradictory rows. In this study, we identified four contradictory rows where some studies covered by the configuration demonstrated improved medication adherence, and others studies covered by the same configuration did *not* demonstrate improvements in adherence. The studies listed here are those that were associated with improved adherence in those rows

^d^An *uppercase letter* in the configuration indicates the BCT was used as part of the study intervention; a *lowercase letter* indicates that the BCT was not used as part of the study intervention. BCTs not listed with either an uppercase or lowercase letter in a configuration were eliminated during the process of logical minimization

^e^Study is uniquely covered by the indicated configuration

### Representation of solutions within and across studies

The remainder of the results section relates the configurations we identified back to the specific studies included in the review to provide examples of how these configurations were represented within the studies. Any individual study covered by a particular configuration may or may not contain other BCTs not explicitly identified by an uppercase or lowercase notation in the solution term. Because the minimization process removes logically redundant terms, the final solutions do not contain a term for all nine BCTs. For example, the set of studies covered by the configuration of “KS” all contain an intervention component to increase *knowledge* AND a component to enhance *self-efficacy*, but any one individual study covered by “KS” may or may not also contain components targeting awareness, attitude, intention formation, action control, maintenance, facilitation, and motivation, all of which “fell out” during the minimization process because their presence or their absence was inconsistently associated with the outcome of improved adherence.

The most empirically relevant configuration we identified was “KS”; studies covered by this configuration included intervention components to increase *knowledge* AND to enhance *self-efficacy*. The 17 studies covered by the “KS” configuration spanned six different clinical conditions (hypertension, depression, diabetes, asthma, congestive heart failure, and hyperlipidemia); all but one targeted only a single chronic condition. “KS” interventions were delivered in-person or by telephone, and also included interventions that were automated, such as the use of computer-generated feedback or mailed educational information. This configuration included several clusters of studies by the same author or research team, though using different study populations. The knowledge component of the “KS” configuration was similar across these 17 studies and was exemplified through a variety of intervention components designed to increase patient knowledge on disease facts (prevalence, symptoms, triggers, pathophysiology), medications available for treatment and side effects of medications, and short- or long-term adverse consequences of poor adherence or no treatment at all.

In the 17 “KS” studies, *knowledge* components were coupled with techniques to raise *self-efficacy*, specifically information and skills needed to overcome barriers to adherence, although the specific self-efficacy techniques used by study interventions varied. These techniques included skills training [17–21], problem solving skills and coping skills [21, 22], and counseling or aids to enhance self-management behaviors and increase self-efficacy to self-manage [22–26]. Some of the studies used theory-based interventions; for example, a highly structured depression treatment program used brief psychotherapy based on Bandura’s social cognitive theory and several social learning theories [21]. Several interventions were based on the transtheoretical model [27]; two of these studies by the same author included stage-matched, computer-generated information reports based on participant responses to a baseline assessment [28, 29]. Similarly, one study used telephone care managers combined with a workbook designed for behavioral activation to support long-term self-management and self-care for patients with depression; the self-efficacy component is exemplified by a focus on identifying and challenging negative thoughts [30]. One study, based on the theory of planned behavior, used a cardiovascular nurse to provide education and counseling for patients with congestive heart failure; the self-efficacy component included skills needed to overcome barriers to adherence [31]. Another study used tailored interactive voice response technology to deliver a behavioral intervention based on the health belief model, social cognitive theory, and self-regulation theory to increase adherence to statin medication [32]. In this intervention, baseline patient measures of knowledge and self-efficacy, in addition to other baseline measures, were used to provide highly tailored feedback to study participants to enhance both knowledge and self-efficacy. One study, based on protection motivation theory, was designed to influence both asthma knowledge and asthma self-efficacy, as both have been associated with adherence behavior [33].

Four studies were covered by the configuration “fG”; these interventions did NOT have a *facilitation* component but did include a *motivational interviewing* component. The “fG” configuration uniquely covered two studies. One of these studies evaluated the use of an automated telephone patient monitoring and counseling approach on adherence to antihypertensive medication and blood pressure control [34]. The *motivational interviewing* component of this study was exemplified by use of motivational counseling messages to improve adherence. The other study uniquely covered by this configuration included a software-based counseling intervention provided by telephone by call center (nonclinical) staff for improving adherence to a specific biological therapy (interferon beta-1a) among patients with multiple sclerosis [35]. The software-based counseling was based on principles of motivational interviewing, as developed by Miller and Rollnick [36]. The absence of a *facilitation* component is exemplified by the automated or semiautomated nature of both interventions, with the absence of continuous professional support, individualizing of regimens, and reducing environmental barriers to adherence.

Two studies were uniquely covered by the “KRFICm” configuration; these studies included components to increase *knowledge* and *awareness*, provide *facilitation*, and increase *intention formation* and use of *action control* but lacked a *maintenance* component. One study involved nurse-led telephone encounters in the Department of Veterans Affairs healthcare system using computer-tailored feedback and home blood pressure monitoring to improve adherence to both antihypertensive regimens and lifestyle behaviors associated with better blood pressure control [37]. The other study was an intervention to prevent readmissions in elderly patients with congestive heart failure [38]. This intervention was mostly delivered face-to-face while patients were still in the hospital, with some follow-up after discharge, using a teaching guide focused on diet and medication adherence.

Three studies, all by the same author, were covered by the “KrFT” configuration, which includes components to increase *knowledge*, provide *facilitation*, and improve *attitude*, but does NOT include an *awareness* component. These studies were similar in intervention design—two were conducted in different populations of patients with depression and diabetes [39, 40] and one study was in patients with depression and hypertension [41]. These studies used an integrated care manager to work with patients and their physicians to individually address factors involved in adherence, based on a conceptual model adapted from Cooper et al. [42].

Only one study was covered by the “kfCm” configuration, which includes an *action control* component, but does not include components to increase *knowledge*, provide *facilitation*, or support maintenance. This study consisted exclusively of daily, 3- to 5-min telephone or video medication reminder calls by a research assistant to community-dwelling patients over age 65 with congestive heart failure [43]. This study exemplifies an effective intervention strategy, despite the absence of components directed at increasing patient knowledge, providing facilitation, or maintenance strategies.

## Discussion

We identified seven different configurations of BCTs that were sufficient for improving medication adherence from a diverse body of evidence. In other words, when one of these configurations is present within an intervention, the intervention demonstrates improved adherence. These configurations accounted for over three quarters of studies demonstrating improved adherence. Further, none of these configurations were identified as sufficient for ineffective interventions. Although the configuration of increasing *knowledge* AND enhancing *self-efficacy* was the most empirically relevant, other sufficient configurations offer alternative pathways to a successful adherence intervention. The configuration increasing knowledge and enhancing self-efficacy was found predominantly among interventions targeting single disease and applicability to adherence interventions designed to target multiple conditions is not known. Perhaps adherence interventions are simplest for staff to implement and for patients to engage in when they involve a single disease. These results generate some hypotheses about what works to improve adherence. These findings could inform future adherence intervention development and testing, specifically the development of intervention features worth subjecting to rigorous comparative effectiveness evaluation.

Overall, we extended the results of the completed review by using QCA to identify configurations of BCTs across clinical conditions, intervention designs, and approaches. Using QCA allowed us to apply a logical process to explore the empirical configurations of BCTs, rather than deconstruct each study into its component BCTs to determine the “net effect” of any one BCT on the outcome of improved adherence. This approach cannot replace a traditional qualitative or quantitative synthesis, but it can complement findings by offering an alternative approach for exploring heterogeneity among interventions and its relationship to outcomes particularly when assumptions required for quantitative probabilistic exploration of heterogeneity (e.g., meta-regression) cannot be met.

Beyond medication adherence interventions, the set-theoretic lens offered by QCA assumes intervention components and the context in which they are delivered are not independent of each other and brings substantive understanding and knowledge into the synthesis. The use of QCA within systematic reviews is new, and we are aware of only a few other applications in this context [44, 45]. These examples are discussed in more detail in our companion article [10].

In conducting the present study, we attempted to apply as many standards of good practice for conducting QCA as was feasible [11]. The greatest challenge we faced was the lack of detail describing intervention components in study publications. Consequently, we erred on the conservative side by assuming that BCTs were absent if it was unclear from the study description. This may have underestimated the number of BCTs used within a study, but we conducted sensitivity analyses assuming that these components were present and found no substantive differences in the findings. In the future, rigorous requirements for intervention description and reporting, the availability of online supplemental materials, and the use of standard taxonomies for describing and cataloging behavioral interventions may mitigate some of the challenges we faced with respect to lack of reported intervention detail.

Our study has several other limitations. Because of the number of BCTs that we elected to include, we had limited diversity. We used some techniques to reduce the number of included BCTs, such as eliminating infrequently used BCTs from the analysis. We also explored the creation of macroconditions by combining single BCTs into one set, but we found substantive experts averse to the idea of coupling several distinct BCTs into one set because of difficulties with interpretation.

Lastly, we used crisp-set calibration because data from the completed review with respect to BCTs and outcomes were abstracted dichotomously. However, this may not reflect the continuum with which BCTs and the outcome may have been represented within studies. The outcome of improved adherence relied on whether at least one measure of adherence demonstrated a statistically significant improvement. The use of statistical significance to determine which studies we deemed “effective” for the QCA carries many of the limitations of the use of statistical significance testing in general, specifically findings due to chance in sampling, failing to find significant effects because of studies that are underpowered, or finding significant effects because of large sample sizes, regardless of whether the magnitude of effect on adherence is clinically meaningful. Lastly, we lacked external standards for establishing quantitative differences in the use of BCTs or adherence outcomes that would have allowed us to use fuzzy-set QCA, an approach that would have allowed more granular distinctions.

## Conclusions

We used a novel method within an existing systematic review to identify several configurations of BCTs among interventions to improve medication adherence in outpatients on chronic medications. Interventions that increase *knowledge* AND enhance *self-efficacy* are sufficient for improving medication adherence; although other configurations of BCTs can also be successful. Using QCA, we were able to capitalize on the intervention heterogeneity within an existing systematic review to uncover patterns that would not have been identified using traditional methods for qualitative or quantitative synthesis. Our findings complement the results from the existing review by offering options for practice or policy and by generating hypotheses for future studies to evaluate the comparative effectiveness and efficiency of different approaches to improving adherence.

## Additional file

Additional file 1: This file provides the truth table for the main analysis, the complex and parsimonious solutions for the main analysis, and results relating to the analysis of outcome complement (studies demonstrating no improvements in medication adherence). (PDF 143 kb)

## Abbreviations

AHRQ: Agency for Healthcare Research and Quality
BCT: behavioral change technique
HIV/AIDS: human immunodeficiency virus/acquired immunodeficiency syndrome
QCA: qualitative comparative analysis
