# Supplementary material for: Identifying configurations of behavior change techniques in effective medication adherence interventions: a qualitative comparative analysis
Source: Syst Rev. 2016 May 4;5:83. doi: 10.1186/s13643-016-0255-z (PMC4875709; doi:10.1186/s13643-016-0255-z)
Supplement: Additional file 1: — This file provides the truth table for the main analysis, the complex and parsimonious solutions for the main analysis, and results relating to the analysis of outcome complement (studies demonstrating no improvements in medication adherence). (PDF 143 kb) [file 13643_2016_255_MOESM1_ESM.pdf]

Additional File 1

Supplemental Analyses

Identifying configurations of behavior change techniques in effective medication adherence interventions: a qualitative comparative analysis

Leila Kahwati, MD, MPH[1], Meera Viswanathan, PhD[1], Carol E. Golin, MD[2], Heather Kane, PhD[1], Megan Lewis, PhD[1], Sara Jacobs, PhD[1]

[1] RTI International, Research Triangle Park, North Carolina

[2] Departments of Medicine and Health Behavior, University of North Carolina, Chapel Hill, North Carolina

Corresponding Author:
Leila Kahwati, MD MPH
RTI International
3040 E. Cornwallis Rd.
Research Triangle Park, NC 27709
Tel: (919) 541-6629
Fax: (9109) 541-6683
lkahwati@rti.org

Appendix A. Truth Table for Main Analysis

Appendix B. Complex and Parsimonious Solutions for Qualitative Comparative Analysis (QCA) of Behavior Change Techniques (BCTs) and Outcome of Improved Medication Adherence

Appendix C. Analyses Relating to Configurations of Behavior Change Techniques (BCTs) in Studies Demonstrating No Improvements in Medication Adherence

References for Appendices

Appendix A

Truth Table for Main Analysis

Table A. Truth Table for Qualitative Comparative Analysis (QCA) of Configurations of Behavior Change Techniques (BCTs) for Improving Medication Adherence. Only rows with empiric cases are shown.

Behavior Change Technique[a]									Number of Studies With Configuration	Consistency (%)	Improved Adherence[b] (Outcome)	Study Citations
Knowledge	Awareness	Attitude	Self-efficacy	Intention Formation	Action Control	Maintenance	Facilitation	Motivational Interviewing				
1	1	0	0	0	1	0	1	0	1	0	0	Pyne et al. 2011[1]
0	0	1	1	1	0	0	0	0	1	100	1	Wolever et al. 2010[2]
1	1	0	0	0	0	0	0	0	5	60	0	Bender et al. 2010[3], Smith et al. 2008[4], Mann et al. 2010[5], Montori et al. 2011[6], Wakefield et al. 2011[7]
1	1	0	1	1	0	0	1	0	1	100	1	Bogner et al. 2010[8]
1	1	0	1	1	0	0	1	1	1	100	1	Wilson et al. 2010[9]
1	0	0	0	0	0	0	1	0	9	44	0	Carter et al. 2009[10], Okeke et al. 2009[11], Waalen et al. 2009[12], Rudd et al. 2009[13], Lee et al. 2006[14], Schnipper et al. 2006[15], Taylor et al. 2003[16], Nietert et al. 2009[17], Solomon et al. 1998[18]
1	1	0	1	0	0	0	0	0	2	100	1	Janson et al. 2009[19], Schaffer et al. 2004[20]
1	1	0	0	0	0	0	1	0	1	0	0	Hunt et al. 2008[21]
1	0	1	0	0	0	0	1	0	1	100	1	Bogner et al. 2008[22]
0	0	0	1	0	1	0	1	0	1	0	0	Pearce et al. 2008[23]
1	1	1	0	1	1	0	1	1	1	100	1	Bosworth et al. 2008[24]
1	0	0	0	0	0	0	0	0	3	33	0	Weymiller et al. 2007[25], Ross et al. 2004[26], Powell et al. 1995[27]
1	0	0	1	0	0	0	1	0	4	100	1	Murray et al. 2007[28], Katon et al. 1996[29], Katon et al. 1995[30], Katon et al. 1999[31]
0	0	0	0	0	0	0	1	0	1	0	0	Sledge et al. 2006[32]
1	1	1	1	0	0	1	0	0	1	100	1	Johnson et al. 2006[33]
1	1	0	0	0	0	0	1	1	1	0	0	Simon et al. 2006[34]
1	1	1	1	0	0	0	0	0	2	100	1	Johnson et al. 2006[35], Wu et al. 2012[36]

Behavior Change Technique[a]									Number of Studies With Configuration	Consistency (%)	Improved Adherence[b] (Outcome)	Study Citations
Knowledge	Awareness	Attitude	Self-efficacy	Intention Formation	Action Control	Maintenance	Facilitation	Motivational Interviewing				
0	0	0	0	1	0	1	0	0	1	0	0	Lin et al. 2006[37]
0	0	0	0	0	0	0	0	1	1	100	1	Berger et al. 2005[38]
1	1	0	0	1	1	1	1	0	1	0	0	Bosworth et al. 2005[39]
1	1	0	1	1	0	1	1	0	1	100	1	Rudd et al. [40]
1	1	0	1	0	0	1	0	0	1	100	1	Janson et al. 2003[41]
1	0	0	0	0	0	1	0	0	1	0	0	Grant et al. 2003[42]
1	0	0	0	0	1	0	0	0	2	50	0	Hoffman et al. 2003[43], Weinberger et al. 2002[44]
1	1	0	0	1	0	1	1	0	1	0	0	Vivian 2002[45]
1	1	0	0	0	1	0	0	0	1	0	0	Guthrie 2001[46]
1	1	0	1	1	1	1	0	1	1	100	1	Katon et al. 2001[47]
0	0	0	0	0	1	0	0	0	1	100	1	Fulmer et al. 1999[48]
1	0	0	1	0	0	0	0	0	1	100	1	Berg et al. 1997[49]
1	1	0	0	1	1	0	1	0	1	100	1	Rich et al. 1996[50]
0	1	0	1	0	0	0	1	0	1	0	0	Schectman et al. 1994[51]
1	1	1	1	1	0	1	0	0	1	100	1	Stacy et al. 2009[52]
1	1	0	0	1	0	0	1	0	2	0	0	Rickles et al. 2005[53], Capoccia et al. 2004[54]
1	1	1	0	0	0	0	0	0	1	0	0	Powers et al. 2011[55]
1	1	0	0	0	0	0	0	1	1	100	1	Friedman et al. 1996[56]
1	0	0	0	1	0	1	1	0	1	100	1	Bogner et al., 2012[57]
1	0	1	1	1	0	0	0	1	1	100	1	Ogedegbe et al. 2012[58]
1	1	1	0	0	0	0	1	1	1	0	0	Solomon et al. 2012[59]
1	0	0	1	1	0	0	1	1	1	100	1	Simon et al. 2004[60]

[a] The presence of the behavior change technique (BCT) is indicated by a "1", the absence of the BCT is indicated by a "0".

[b] A "1" indicates that studies with the configuration of BCTs demonstrate improvements in medication adherence. A "0" indicates that studies with the configuration of BCTs either did not demonstrate improvements in medication adherence, or the studies had contradictory results, with some demonstrating improvements and some not demonstrating improvements.

Appendix B

Complex and Parsimonious Solutions for Qualitative Comparative Analysis of Behavior Change Techniques and Outcome of Improved Medication Adherence

Table B-1. Fourteen Sufficient Configurations of Behavior Change Techniques (BCTs) for Improving Medication Adherence Identified in the Complex Solution[a]

Behavior Change Technique Configuration[b]	Number of Studies Covered	Raw Coverage[c] (%)	Unique Coverage[d] (%)	Consistency[e] (%)	Studies Covered
KRfSicg	6	18	15	100	Schaffer et al. 2004[20]<br>Johnson et al. 2006[35]<br>Johnson et al. 2006[33]<br>Wu et al. 2012[36]<br>Janson et al. 2009[19]<br>Janson et al. 2003[41]
KrSticmg	5	15	15	100	Katon et al. 1995[30]<br>Berg et al. 1997[49]<br>Katon et al. 1999[31]<br>Katon et al. 1996[29]<br>Murray et al. 2007[28]
KRfSTcMg	2	6	3	100	Stacy et al. 2009[52]<br>Johnson et al. 2006[33]
KRFStIcg	1	3	3	100	Rudd et al. 2004[40]
KFStIcmG	2	6	6	100	Wilson et al. 2010[9]<br>Simon et al. 2004[60]
krfstiCmg	1	3	3	100	Fulmer et al. 1999[48]
krfsticmG	1	3	3	100	Berger et al. 2005[38]
krfSTIcmg	1	3	3	100	Wolever et al. 2010[2]
KrFsTicmg	3	9	9	100	Bogner et al. 2008[22]<br>Bogner et al. 2012[57]<br>Bogner et al. 2010[8]
KRfsticmG	1	3	3	100	Friedman et al. 1996[56]
KRFstICmg	1	3	3	100	Rich et al. 1996[50]
KrfSTIcmG	1	3	3	100	Ogedegbe et al. 2012[58]
KRfStICMG	1	3	3	100	Katon et al. 2001[47]
KRFsTICmG	1	3	3	100	Bosworth et al. 2008[24]
Solution Consistency (%) 100					
Solution Coverage (%) 76					
Overlapping Coverage (%) 6					

[a] The complex solution represents the logical minimization of only the truth table rows that are covered by studies with the outcome present (N=19 rows). Logical remainders (empty truth table rows) are not included in the process of logical minimization.

[b] An uppercase letter in the configuration indicates the BCT was used as part of the study intervention; a lowercase letter indicates the BCT was not used as part of the study intervention. BCTs not listed in the configuration were eliminated during the process of logical minimization.

[c] Raw coverage is determined by dividing the number of studies covered by the configuration by the number of studies demonstrating improved adherence (34 in this analysis). Coverage can range from 0 to 100%.

[d] Unique coverage is determined by dividing the number of studies that are only covered by the configuration by the number of studies demonstrating improved adherence (34 in this analysis). Coverage can range from 0 to 100%.

[e] Consistency is determined by dividing the number of studies in the outcome set covered by the configuration by the number of studies covered by the configuration. Consistency can range from 0 to 100%. A consistency of 100% indicates that all studies covered by the configuration were also in the outcome set (i.e., had improved adherence).

Table B-2. Five Sufficient Configurations of Behavior Change Techniques (BCTS) for Improving Medication Adherence Identified in the Parsimonious Solution[a]

**Behavior Change Technique Configuration**[b]	**Number of Studies Covered**	**Raw Coverage**[c] **(%)**	**Unique Coverage**[d] **(%)**	**Consistency**[e] **(%)**	**Studies Covered**
KS	17	50	44	100	Murray et al. 2007[28]<br>Simon et al. 2004[60]<br>Katon et al. 1996[29]<br>Katon et al. 1999[31]<br>Berg et al. 1997[49]<br>Janson et al. 2003[41]<br>Rudd et al. 2004[40]<br>Janson et al. 2009[19]<br>Wu et al. 2012[36]<br>Johnson et al. 2006[33]<br>Johnson et al. 2006[35]<br>Stacy et al. 2009[52]<br>Ogedegbe et al. 2012[58]<br>Wilson et al. 2010[9]<br>Katon et al. 1995[30]<br>Katon et al. 2001[47]<br>Schaffer et al. 2004[20]
rT	5	15	9	100	Bogner et al. 2008[22]<br>Bogner et al. 2012[57]<br>Wolever et al. 2010[2]<br>Ogedegbe et al. 2012[58]<br>Bogner et al. 2010[8]
fG	4	12	3	100	Katon et al. 2001[47]<br>Ogedegbe et al. 2012[58]<br>Berger et al. 2005[38]<br>Friedman et al. 1996[56]
kfm	3	9	3	100	Wolever et al. 2010[2]<br>Fulmer et al. 1999[48]<br>Berger et al. 2005[38]
ICM	2	6	6	100	Bosworth et al. 2008[24]<br>Rich et al. 1996[50]
Solution Consistency(%) 100					
Solution Coverage (%) 76					
Overlapping Coverage (%) 11					

[a] The parsimonious solution represents the automated, logical minimization of all truth table rows (N=512 rows) with simplifying assumptions about logical remainders made by the software to result in the most parsimonious number of solution terms. The plausibility of the simplifying assumptions made about logical remainders is not evaluated.

[b] An uppercase letter in the configuration indicates the BCT was used as part of the study intervention; a lowercase letter in the configuration indicates the BCT was not used as part of the study intervention. BCTs not listed in the configuration were eliminated during the process of logical minimization.

[c] Raw coverage is determined by dividing the number of studies covered by the configuration by the number of studies demonstrating improved adherence (34 in this analysis). Coverage can range from 0 to 100%.

[d] Unique coverage is determined by dividing the number of studies that are only covered by the configuration by the number of studies demonstrating improved adherence (34 in this analysis). Coverage can range from 0 to 100%.

[e] Consistency is determined by dividing the number of studies in the outcome set covered by the configuration by the number of studies covered by the configuration. Consistency can range from 0 to 100%. A consistency of 100% indicates that all studies covered by the configuration are also in the outcome set (i.e., had improved adherence).

Appendix C

Analyses Relating to Configurations of Behavior Change Techniques in Studies Demonstrating *No* Improvements in Medication Adherence

Table C-1. Necessity and Sufficiency for Individual Behavior Change Techniques (BCTs) Used Within Studies Demonstrating *NO* Improvements in Medication Adherence

			**Necessity**	**Sufficiency**
**Behavior Change Technique (Abbreviation for analysis[a])**	**Number of Studies with BCT**	**Number of Studies with BCT from Among Those Studies Demonstrating *No* Improvements in Adherence (N=26)[b]**	**Of Studies Demonstrating *No* Improvements in Adherence(N=26)[b], Percent with BCT**	**Of studies with BCT, Percent that Demonstrate *No* Improvements in Adherence**
Knowledge (K)	53	22	85	42
Facilitation (F)	32	16	62	50
Awareness (R)	29	14	54	48
Self-efficacy (S)	20	2	8	10
Intention formation (I)	15	5	19	33
Action control (C)	10	5	19	50
Attitude (T)	11	2	8	18
Maintenance (M)	10	4	15	40
Motivational interviewing (G)	9	2	8	22

[a] Abbreviations are used in the presentation of results.
[b] 26 studies demonstrated no improvements in medication adherence out of a total of 60 included studies.

Table C-2. Eight Sufficient Configurations of Behavior Change Techniques (BCTs) Identified in the Parsimonious Solution[a] for the Outcome of *NO* Improvements in Medication Adherence

**Behavior Change Technique Configuration[b]**	**Number of Studies Covered**	**Raw Coverage[c] (%)**	**Unique Coverage[d] (%)**	**Consistency[e] (%)**	**Studies Covered**
kF	3	12	12	100	Pearce et al. 2008[23]<br>Sledge et al. 2006[32]<br>Schectman et al. 1994[51]
RiC	2	8	8	100	Pyne et al. 2011[1]<br>Guthrie et al. 2001[46]
sM	4	15	12	100	Lin et al. 2006[37]<br>Bosworth et al. 2005[39]<br>Grant et al. 2003[42]<br>Vivian et al. 2002[45]
RFsc	6	23	15	100	Hunt et al. 2008[21]<br>Simon et al. 2006[34]<br>Vivian et al. 2002[45]<br>Rickles et al. 2005[53]<br>Capoccia et al. 2004[54]<br>Solomon et al. 2012[59]
sTf[f]	1	4	0	100	Powers et al. 2011[55]
RsTg[f]	1	4	0	100	Powers et al. 2011[55]
RsTc[f]	2	8	0	100	Powers et al. 2011[55]<br>Solomon et al. 2012[59]
RsTi[f]	2	8	0	100	Powers et al. 2011[55]<br>Solomon et al. 2012[59]
Solution Consistency (%) 100					
Solution Coverage (%) 58					
Overlapping Coverage (%) 35					

[a] The parsimonious solution represents the automated, logical minimization of all truth table rows (N=512 rows) with simplifying assumptions about logical remainders made by the software to result in the most parsimonious number of solution terms. The plausibility of the simplifying assumptions made about logical remainders is not evaluated.

[b] An uppercase letter in the configuration indicates the BCT was used as part of the study intervention; a lowercase letter in the configuration indicates the BCT was not used as part of the study intervention. BCTs not listed in the configuration were eliminated during the process of logical minimization.

[c] Raw coverage is determined by dividing the number of studies covered by the configuration by the number of studies demonstrating *no* improved adherence (26 in this analysis). Coverage can range from 0 to 100%.

[d] Unique coverage is determined by dividing the number of studies that are only covered by the configuration by the number of studies demonstrating *no* improved adherence (26 in this analysis). Coverage can range from 0 to 100%.

[e] Consistency is determined by dividing the number of studies in the outcome set covered by the configuration by the number of studies covered by the configuration. Consistency can range from 0 to 100%. A consistency of 100% indicates that all studies covered by the configuration were also in the outcome set (i.e., had *no* improved adherence).

[f] These configurations are logically redundant, the parsimonious solution can have as few as five sufficient conditions if one of these configurations is preferred over any of the others, though we could identify no substantive reason to prefer one over any of the others.

Table C-3. Ten Sufficient Configurations of Behavior Change Techniques (BCTs) Identified in the Complex Solution[a] for the Outcome of *NO* Improvement in Medication Adherence

**Behavior Change Technique Configuration[b]**	**Number of Studies Covered**	**Raw Coverage[c] (%)**	**Unique Coverage[d] (%)**	**Consistency[e] (%)**	**Studies Covered**
KRtsiCmg	2	8	8	100	Pyne et al. 2011[1]<br>Guthrie et al. 2001[46]
KRtscmFg	3	2	12	100	Hunt et al. 2008[21]<br>Rickles et al. 2005[53]<br>Capoccia et al. 2004[54]
KRsicmFG	2	8	8	100	Simon et al. 2006[34]<br>Solomon et al. 2012[59]
KRtsIMFg	2	8	8	100	Bosworth et al. 2005[39]<br>Vivian et al. 2002[45]
krtsicmFg	1	4	4	100	Sledge et al. 2006[32]
KrtsicMfg	1	4	4	100	Grant et al. 2003[42]
krtsIcMfg	1	4	4	100	Lin et al. 2006[37]
KRTsicmfg	1	4	4	100	Powers et al. 2011[55]
kRtSicmFg	1	4	4	100	Schectman et al. 1994[51]
krtSicmFg	1	4	4	100	Pearce et al. 2008[23]
Solution Consistency (%) 100					
Solution Coverage (%) 58					
Overlapping Coverage (%) 0					

[a] The complex solution represents the logical minimization of truth table rows that are covered by studies with the outcome absent (N=18 rows). Logical remainders (empty truth table rows) are not included in the process of logical minimization.

[b] An uppercase letter in the configuration indicates the BCT was used as part of the study intervention; a lowercase letter indicates the BCT was not used as part of the study intervention. BCTs not listed in the configuration were eliminated during the process of logical minimization.

[c] Raw coverage is determined by dividing the number of studies covered by the configuration by the number of studies demonstrating *no* improved adherence (26 in this analysis). Coverage can range from 0 to 100%.

[d] Unique coverage is determined by dividing the number of studies that are only covered by the configuration by the number of studies demonstrating *no* improved adherence (26 in this analysis). Coverage can range from 0 to 100%.

[e] Consistency is determined by dividing the number of studies in the outcome set covered by the configuration by the number of studies covered by the configuration. Consistency can range from 0 to 100%. A consistency of 100% indicates that all studies covered by the configuration were also in the outcome set (i.e., had *no* improved adherence).

Table C-4. Seven Sufficient Configurations of Behavior Change Techniques (BCTs) Identified in the Intermediate Solution[a] for the Outcome of *NO* Improvement in Medication Adherence

**Behavior Change Technique Configuration[b]**	**Number of Studies Covered**	**Raw Coverage[c] (%)**	**Unique Coverage[d] (%)**	**Consistency[e] (%)**	**Studies Covered**
stMg	4	15	12	100	Lin et al. 2006[37]<br>Grant et al. 2003[42]<br>Bosworth et al. 2005[39]<br>Vivian et al. 2002[45]
RFsicm	3	12	8	100	Simon et al. 2006[34]<br>Hunt et al. 2008[21]<br>Solomon et al. 2012[59]
RFstcg	4	15	8	100	Hunt et al. 2008[21]<br>Vivian et al. 2002[45]<br>Rickles et al. 2005[53]<br>Capoccia et al. 2004[54]
RCistg	2	8	8	100	Pyne et al. 2011[1]<br>Guthrie et al. 2001[46]
krFtimg	2	8	4	100	Sledge et al. 2006[32]<br>Pearce et al. 2008[23]
kFitcmg	2	8	4	100	Pearce et al. 2008[23]<br>Schectman et al. 1994[51]
sTicfmg	1	4	4	100	Powers et al. 2011[55]
Solution Consistency (%) 100					
Solution Coverage (%) 58					
Overlapping Coverage (%) 0					

[a] The intermediate solution represents the logical minimization of all truth table rows, based on researcher-directed simplifying assumptions about logical remainders to result in a more parsimonious solution than the complex solution. For the logical remainders in this analysis, we assumed the absence of a BCT would lead to the outcome of *no* improved adherence.

[b] An uppercase letter in the configuration indicates the BCT was used as part of the study intervention; a lowercase letter in the configuration indicates the BCT was not used as part of the study intervention. BCTs not listed in the configuration were eliminated during the process of logical minimization.

[c] Raw coverage is determined by dividing the number of studies covered by the configuration by the number of studies demonstrating *no* improved adherence (26 in this analysis). Coverage can range from 0 to 100%.

[d] Unique coverage is determined by dividing the number of studies that are only covered by the configuration by the number of studies demonstrating *no* improved adherence (26 in this analysis). Coverage can range from 0 to 100%.

[e] Consistency is determined by dividing the number of studies in the outcome set covered by the configuration by the number of studies covered by the configuration. Consistency can range from 0 to 100%. A consistency of 100% indicates that all studies covered by the configuration were also in the outcome set (i.e., had *no* improved adherence).

References

1. Pyne JM, Fortney JC, Curran GM, Tripathi S, Atkinson JH, Kilbourne AM et al. Effectiveness of collaborative care for depression in human immunodeficiency virus clinics. Arch Intern Med. 2011;171(1):23-31. doi:10.1001/archinternmed.2010.395.

2. Wolever RQ, Dreusicke M, Fikkan J, Hawkins TV, Yeung S, Wakefield J et al. Integrative health coaching for patients with type 2 diabetes: a randomized clinical trial. Diabetes Educ. 2010;36(4):629-39. doi:10.1177/0145721710371523.

3. Bender BG, Apter A, Bogen DK, Dickinson P, Fisher L, Wamboldt FS et al. Test of an interactive voice response intervention to improve adherence to controller medications in adults with asthma. J Am Board Fam Med. 2010;23(2):159-65. doi:10.3122/jabfm.2010.02.090112.

4. Smith DH, Kramer JM, Perrin N, Platt R, Roblin DW, Lane K et al. A randomized trial of direct-to-patient communication to enhance adherence to beta-blocker therapy following myocardial infarction. Arch Intern Med. 2008;168(5):477-83; discussion 83; quiz 47. doi:10.1001/archinternmed.2007.132.

5. Mann DM, Ponieman D, Montori VM, Arciniega J, McGinn T. The Statin Choice decision aid in primary care: a randomized trial. Patient Educ Couns. 2010;80(1):138-40. doi:10.1016/j.pec.2009.10.008.

6. Montori VM, Shah ND, Pencille LJ, Branda ME, Van Houten HK, Swiglo BA et al. Use of a decision aid to improve treatment decisions in osteoporosis: the osteoporosis choice randomized trial. Am J Med. 2011;124(6):549-56. doi:10.1016/j.amjmed.2011.01.013.

7. Wakefield BJ, Holman JE, Ray A, Scherubel M, Adams MR, Hillis SL et al. Effectiveness of home telehealth in comorbid diabetes and hypertension: a randomized, controlled trial. Telemed J E Health. 2011;17(4):254-61. doi:10.1089/tmj.2010.0176.

8. Bogner HR, de Vries HF. Integrating type 2 diabetes mellitus and depression treatment among African Americans: a randomized controlled pilot trial. Diabetes Educ. 2010;36(2):284-92. doi:10.1177/0145721709356115.

9. Wilson SR, Strub P, Buist AS, Knowles SB, Lavori PW, Lapidus J et al. Shared treatment decision making improves adherence and outcomes in poorly controlled asthma. Am J Respir Crit Care Med. 2010;181(6):566-77. doi:10.1164/rccm.200906-0907OC.

10. Carter BL, Ardery G, Dawson JD, James PA, Bergus GR, Doucette WR et al. Physician and pharmacist collaboration to improve blood pressure control. Arch Intern Med. 2009;169(21):1996-2002. doi:10.1001/archinternmed.2009.358.

11. Okeke CO, Quigley HA, Jampel HD, Ying GS, Plyler RJ, Jiang Y et al. Interventions improve poor adherence with once daily glaucoma medications in electronically monitored patients. Ophthalmology. 2009;116(12):2286-93. doi:10.1016/j.ophtha.2009.05.026.

12. Waalen J, Bruning AL, Peters MJ, Blau EM. A telephone-based intervention for increasing the use of osteoporosis medication: a randomized controlled trial. Am J Manag Care. 2009;15(8):e60-70.

13. Rudd RE, Blanch DC, Gall V, Chibnik LB, Wright EA, Reichmann W et al. A randomized controlled trial of an intervention to reduce low literacy barriers in inflammatory arthritis management. Patient Educ Couns. 2009;75(3):334-9. doi:10.1016/j.pec.2009.03.001.

14. Lee JK, Grace KA, Taylor AJ. Effect of a pharmacy care program on medication adherence and persistence, blood pressure, and low-density lipoprotein cholesterol: a randomized controlled trial. JAMA. 2006;296(21):2563-71. doi:10.1001/jama.296.21.joc60162.

15. Schnipper JL, Kirwin JL, Cotugno MC, Wahlstrom SA, Brown BA, Tarvin E et al. Role of pharmacist counseling in preventing adverse drug events after hospitalization. Arch Intern Med. 2006;166(5):565-71. doi:10.1001/archinte.166.5.565.

16. Taylor CT, Byrd DC, Krueger K. Improving primary care in rural Alabama with a pharmacy initiative. Am J Health Syst Pharm. 2003;60(11):1123-9.

17. Nietert PJ, Tilley BC, Zhao W, Edwards PF, Wessell AM, Mauldin PD et al. Two pharmacy interventions to improve refill persistence for chronic disease medications: a randomized, controlled trial. Med Care. 2009;47(1):32-40. doi:10.1097/MLR.0b013e3181808c17.

18. Solomon DK, Portner TS, Bass GE, Gourley DR, Gourley GA, Holt JM et al. Clinical and economic outcomes in the hypertension and COPD arms of a multicenter outcomes study. J Am Pharm Assoc (Wash). 1998;38(5):574-85.

19. Janson SL, McGrath KW, Covington JK, Cheng SC, Boushey HA. Individualized asthma self-management improves medication adherence and markers of asthma control. J Allergy Clin Immunol. 2009;123(4):840-6. doi:10.1016/j.jaci.2009.01.053.

20. Schaffer SD, Tian L. Promoting adherence: effects of theory-based asthma education. Clin Nurs Res. 2004;13(1):69-89. doi:10.1177/1054773803259300.

21. Hunt JS, Siemienczuk J, Pape G, Rozenfeld Y, MacKay J, LeBlanc BH et al. A randomized controlled trial of team-based care: impact of physician-pharmacist collaboration on uncontrolled hypertension. J Gen Intern Med. 2008;23(12):1966-72. doi:10.1007/s11606-008-0791-x.

22. Bogner HR, de Vries HF. Integration of depression and hypertension treatment: a pilot, randomized controlled trial. Ann Fam Med. 2008;6(4):295-301. doi:10.1370/afm.843.

23. Pearce KA, Love MM, Shelton BJ, Schoenberg NE, Williamson MA, Barron MA et al. Cardiovascular risk education and social support (CaRESS): report of a randomized controlled trial from the Kentucky Ambulatory Network (KAN). J Am Board Fam Med. 2008;21(4):269-81. doi:10.3122/jabfm.2008.04.080007.

24. Bosworth HB, Olsen MK, Neary A, Orr M, Grubber J, Svetkey L et al. Take Control of Your Blood Pressure (TCYB) study: a multifactorial tailored behavioral and educational intervention for achieving blood pressure control. Patient Educ Couns. 2008;70(3):338-47. doi:10.1016/j.pec.2007.11.014.

25. Weymiller AJ, Montori VM, Jones LA, Gafni A, Guyatt GH, Bryant SC et al. Helping patients with type 2 diabetes mellitus make treatment decisions: statin choice randomized trial. Arch Intern Med. 2007;167(10):1076-82. doi:10.1001/archinte.167.10.1076.

26. Ross SE, Moore LA, Earnest MA, Wittevrongel L, Lin CT. Providing a web-based online medical record with electronic communication capabilities to patients with congestive heart failure: randomized trial. J Med Internet Res. 2004;6(2):e12. doi:10.2196/jmir.6.2.e12.

27. Powell KM, Edgren B. Failure of educational videotapes to improve medication compliance in a health maintenance organization. Am J Health Syst Pharm. 1995;52(20):2196-9.

28. Murray MD, Young J, Hoke S, Tu W, Weiner M, Morrow D et al. Pharmacist intervention to improve medication adherence in heart failure: a randomized trial. Ann Intern Med. 2007;146(10):714-25.

29. Katon W, Robinson P, Von Korff M, Lin E, Bush T, Ludman E et al. A multifaceted intervention to improve treatment of depression in primary care. Arch Gen Psychiatry. 1996;53(10):924-32.

30. Katon W, Von Korff M, Lin E, Walker E, Simon GE, Bush T et al. Collaborative management to achieve treatment guidelines. Impact on depression in primary care. JAMA. 1995;273(13):1026-31.

31. Katon W, Von Korff M, Lin E, Simon G, Walker E, Unutzer J et al. Stepped collaborative care for primary care patients with persistent symptoms of depression: a randomized trial. Arch Gen Psychiatry. 1999;56(12):1109-15.

32. Sledge WH, Brown KE, Levine JM, Fiellin DA, Chawarski M, White WD et al. A randomized trial of primary intensive care to reduce hospital admissions in patients with high utilization of inpatient services. Dis Manag. 2006;9(6):328-38. doi:10.1089/dis.2006.9.328.

33. Johnson SS, Driskell MM, Johnson JL, Prochaska JM, Zwick W, Prochaska JO. Efficacy of a transtheoretical model-based expert system for antihypertensive adherence. Dis Manag. 2006;9(5):291-301. doi:10.1089/dis.2006.9.291.

34. Simon GE, Ludman EJ, Operskalski BH. Randomized trial of a telephone care management program for outpatients starting antidepressant treatment. Psychiatr Serv. 2006;57(10):1441-5. doi:10.1176/appi.ps.57.10.1441.

35. Johnson SS, Driskell MM, Johnson JL, Dyment SJ, Prochaska JO, Prochaska JM et al. Transtheoretical model intervention for adherence to lipid-lowering drugs. Dis Manag. 2006;9(2):102-14. doi:10.1089/dis.2006.9.102.

36. Wu JR, Corley DJ, Lennie TA, Moser DK. Effect of a medication-taking behavior feedback theory-based intervention on outcomes in patients with heart failure. J Card Fail. 2012;18(1):1-9. doi:10.1016/j.cardfail.2011.09.006.

37. Lin EH, Katon W, Rutter C, Simon GE, Ludman EJ, Von Korff M et al. Effects of enhanced depression treatment on diabetes self-care. Ann Fam Med. 2006;4(1):46-53. doi:10.1370/afm.423.

38. Berger BA, Liang H, Hudmon KS. Evaluation of software-based telephone counseling to enhance medication persistency among patients with multiple sclerosis. J Am Pharm Assoc (2003). 2005;45(4):466-72.

39. Bosworth HB, Olsen MK, Gentry P, Orr M, Dudley T, McCant F et al. Nurse administered telephone intervention for blood pressure control: a patient-tailored multifactorial intervention. Patient Educ Couns. 2005;57(1):5-14. doi:10.1016/j.pec.2004.03.011.

40. Rudd P, Miller NH, Kaufman J, Kraemer HC, Bandura A, Greenwald G et al. Nurse management for hypertension. A systems approach. Am J Hypertens. 2004;17(10):921-7. doi:10.1016/j.amjhyper.2004.06.006.

41. Janson SL, Fahy JV, Covington JK, Paul SM, Gold WM, Boushey HA. Effects of individual self-management education on clinical, biological, and adherence outcomes in asthma. Am J Med. 2003;115(8):620-6.

42. Grant RW, Devita NG, Singer DE, Meigs JB. Improving adherence and reducing medication discrepancies in patients with diabetes. Ann Pharmacother. 2003;37(7-8):962-9.

43. Hoffman L, Enders J, Luo J, Segal R, Pippins J, Kimberlin C. Impact of an antidepressant management program on medication adherence. Am J Manag Care. 2003;9(1):70-80.

44. Weinberger M, Murray MD, Marrero DG, Brewer N, Lykens M, Harris LE et al. Effectiveness of pharmacist care for patients with reactive airways disease: a randomized controlled trial. JAMA. 2002;288(13):1594-602.

45. Vivian EM. Improving blood pressure control in a pharmacist-managed hypertension clinic. Pharmacotherapy. 2002;22(12):1533-40.

46. Guthrie RM. The effects of postal and telephone reminders on compliance with pravastatin therapy in a national registry: results of the first myocardial infarction risk reduction program. Clin Ther. 2001;23(6):970-80.

47. Katon W, Rutter C, Ludman EJ, Von Korff M, Lin E, Simon G et al. A randomized trial of relapse prevention of depression in primary care. Arch Gen Psychiatry. 2001;58(3):241-7.

48. Fulmer TT, Feldman PH, Kim TS, Carty B, Beers M, Molina M et al. An intervention study to enhance medication compliance in community-dwelling elderly individuals. J Gerontol Nurs. 1999;25(8):6-14.

49. Berg J, Dunbar-Jacob J, Sereika SM. An evaluation of a self-management program for adults with asthma. Clin Nurs Res. 1997;6(3):225-38.

50. Rich MW, Gray DB, Beckham V, Wittenberg C, Luther P. Effect of a multidisciplinary intervention on medication compliance in elderly patients with congestive heart failure. Am J Med. 1996;101(3):270-6.

51. Schectman G, Hiatt J, Hartz A. Telephone contacts do not improve adherence to niacin or bile acid sequestrant therapy. Ann Pharmacother. 1994;28(1):29-35.

52. Stacy JN, Schwartz SM, Ershoff D, Shreve MS. Incorporating tailored interactive patient solutions using interactive voice response technology to improve statin adherence: results of a randomized clinical trial in a managed care setting. Popul Health Manag. 2009;12(5):241-54. doi:10.1089/pop.2008.0046.

53. Rickles NM, Svarstad BL, Statz-Paynter JL, Taylor LV, Kobak KA. Pharmacist telemonitoring of antidepressant use: effects on pharmacist-patient collaboration. J Am Pharm Assoc (2003). 2005;45(3):344-53.

54. Capoccia KL, Boudreau DM, Blough DK, Ellsworth AJ, Clark DR, Stevens NG et al. Randomized trial of pharmacist interventions to improve depression care and outcomes in primary care. Am J Health Syst Pharm. 2004;61(4):364-72.

55. Powers BJ, Danus S, Grubber JM, Olsen MK, Oddone EZ, Bosworth HB. The effectiveness of personalized coronary heart disease and stroke risk communication. Am Heart J. 2011;161(4):673-80.

56. Friedman RH, Kazis LE, Jette A, Smith MB, Stollerman J, Torgerson J et al. A telecommunications system for monitoring and counseling patients with hypertension. Impact on medication adherence and blood pressure control. Am J Hypertens. 1996;9(4 Pt 1):285-92. doi:0895706195003533 [pii].

57. Bogner HR, Morales KH, de Vries HF, Cappola AR. Integrated management of type 2 diabetes mellitus and depression treatment to improve medication adherence: arandomized controlled trial. Ann Fam Med. 2012;10(1):15-22. doi:10.1370/afm.1344.

58. Ogedegbe GO, Boutin-Foster C, Wells MT, Allegrante JP, Isen AM, Jobe JB et al. A randomized controlled trial of positive-affect intervention and medication adherence in hypertensive African Americans. Arch Intern Med. 2012;172(4):322-6. doi:10.1001/archinternmed.2011.1307.

59. Solomon DH, Iversen MD, Avorn J, Gleeson T, Brookhart MA, Patrick AR et al. Osteoporosis telephonic intervention to improve medication regimen adherence: a large, pragmatic, randomized controlled trial. Arch Intern Med. 2012;172(6):477-83. doi:10.1001/archinternmed.2011.1977.

60. Simon GE, Ludman EJ, Tutty S, Operskalski B, Von Korff M. Telephone psychotherapy and telephone care management for primary care patients starting antidepressant treatment: a randomized controlled trial. JAMA. 2004;292(8):935-42. doi:10.1001/jama.292.8.935.
